# Supplementary material for: The Validity of Benchmark Dose Limit Analysis for Estimating Permissible Accumulation of Cadmium
Source: Int J Environ Res Public Health. 2022 Nov 25;19(23):15697. doi: 10.3390/ijerph192315697 (PMC9736539; doi:10.3390/ijerph192315697)
Supplement: Supplementary file 1 [file ijerph-19-15697-s001.zip › ijerph-1939724-supplementary.pdf]

Supplemental Material: The Validity of Benchmark Dose Limit Analysis for Estimating Permissible Accumulation of Cadmium

Soisungwan Satarug, David A. Vesey, Glenda C. Gobe, and Aleksandra Buha Đorđević

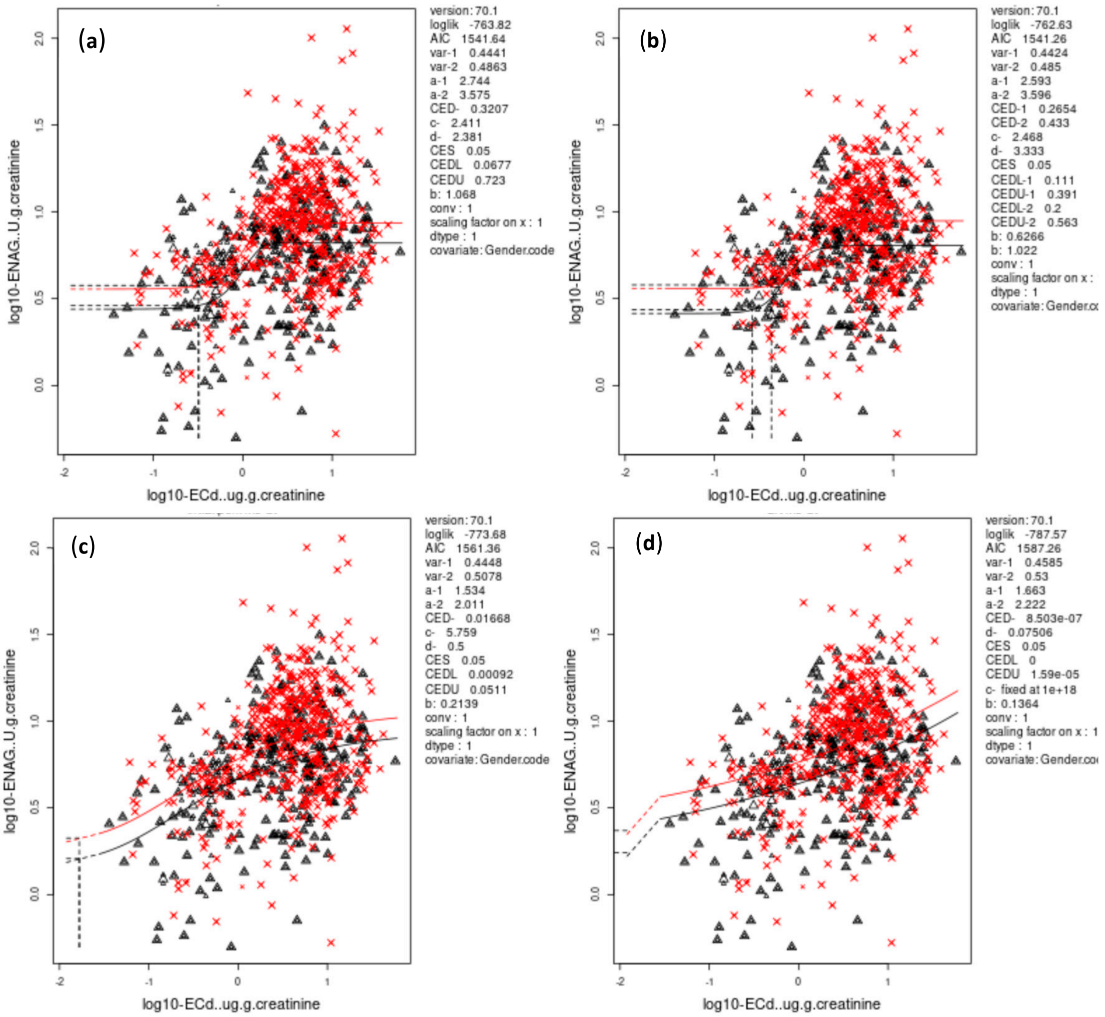

(e)

Continuous Endpoint	BMDL	BMDU	U/L
5% Increase of E_{NAG}/E_{cr}			
Men	0.060	0.504	8.44
Women	0.069	0.537	7.79

BMDL and BMDU values of E_{Cd}/E_{cr} were as µg/g creatinine. CI, Confidence interval; U/L, BMDU/BMDL ratio.

Figure S1. Continuous dose-response analysis of E_{Cd}/E_{cr} and E_{NAG}/E_{cr}. Datasets were fitted to 4 dose-response models; an exponential model (**a**), Hill model (**b**), an inverse exponential model (**c**), and natural logarithmic model (**d**). Model averaging BMDL/BMDU values of E_{Cd}/E_{cr} associated with a 5% increase in E_{NAG}/E_{cr} (**e**).

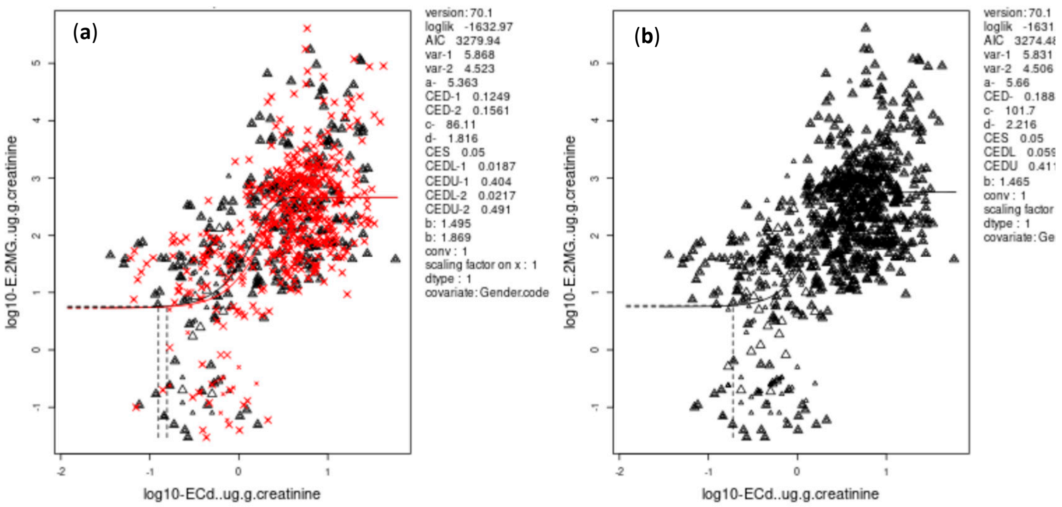

(c)

Continuous Endpoint	BMDL	BMDU	U/L
5% Increase of $E_{\beta 2MG}/E_{cr}$			
Men	0.019	0.411	22
Women	0.022	0.491	23

BMDL and BMDU values of E_{Cd}/E_{cr} were as µg/g creatinine. CI, Confidence interval; U/L, BMDU/BMDL ratio.

Figure S2. Continuous dose-response analysis of E_{Cd}/E_{cr} and $E_{\beta 2MG}/E_{cr}$. Datasets were fitted to an exponential model (**a**), and Hill model (**b**). Model averaging BMDL/BMDU values of E_{Cd}/E_{cr} associated with a 5% increase in $E_{\beta 2MG}/E_{cr}$ (**c**).

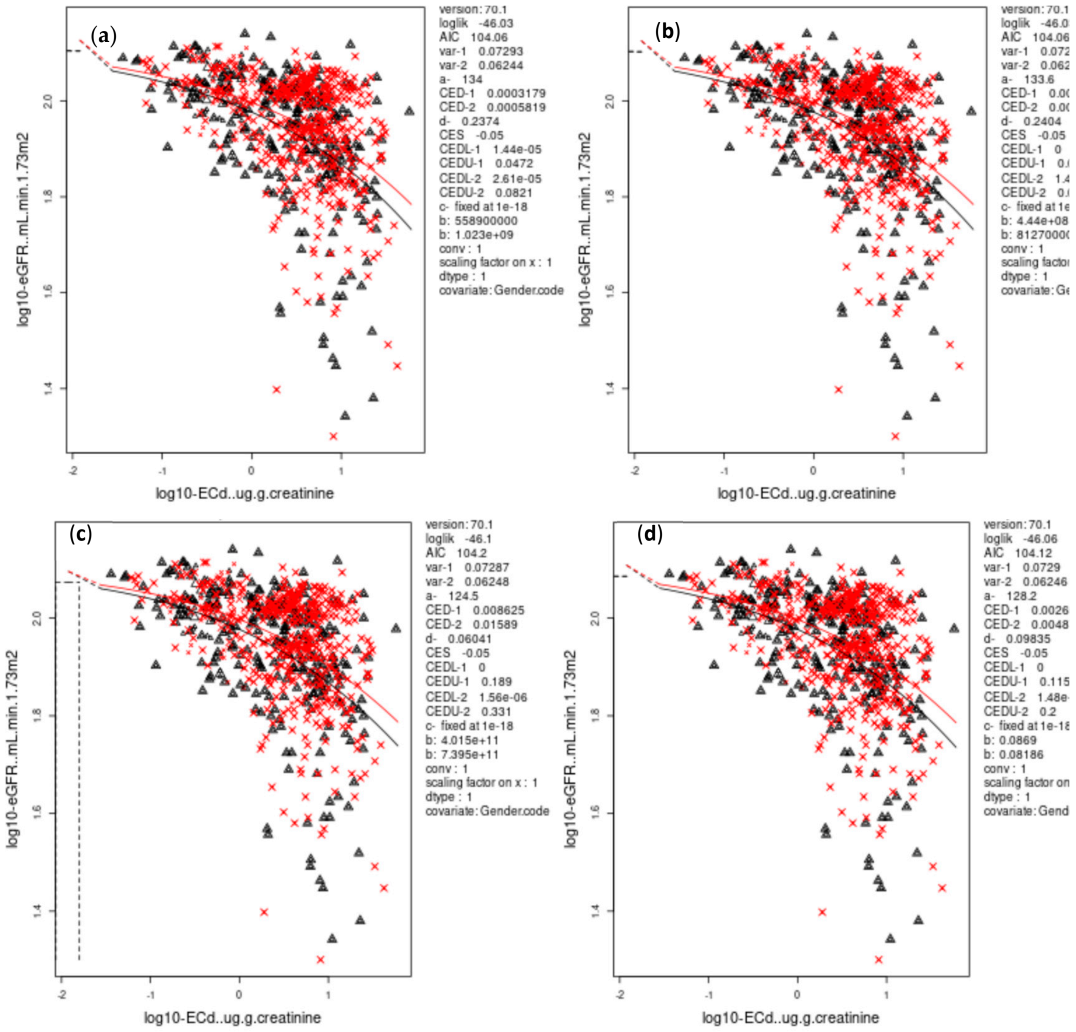

(f)

Continuous Endpoint	BMDL	BMDU	U/L
5% Decrease of eGFR			
Men	1.57×10^{-6}	0.427	2.7×10^{5}
Women	1.88×10^{-5}	0.495	2.6×10^{4}

BMDL and BMDU values of E_{Cd}/E_{cr} were as µg/g creatinine.
CI, confidence interval; U/L, BMDU/BMDL ratio.

Figure S3. Continuous dose-response analysis of E_{Cd}/E_{cr} and eGFR. Datasets were fitted to 4 dose-response models including, an exponential model (a), Hill model (b), an inverse exponential model (c), and natural logarithmic model (d). Model averaging BMDL/BMDU values of E_{Cd}/E_{cr} associated with a 5% decrease in eGFR (e).

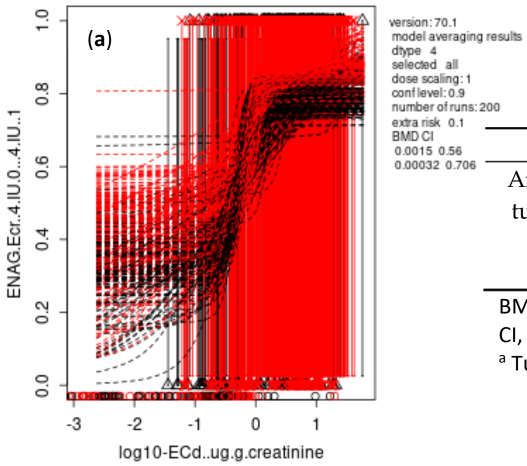

(b)

Quantal endpoint	BMDL	BMDU	U/L
An increase in prevalence of tubular cell injury [a] by 10%.			
Men	1.48×10^{-3}	0.560	378
Women	3.17×10^{-4}	0.706	2227

BMDL and BMDU values of E_{Cd}/E_{cr} were as µg/g creatinine.
CI, confidence interval; U/L, BMDU/BMDL ratio.
[a] Tubular cell injury was defined as $E_{NAG}/E_{cr} \geq 4$ U/g creatinine.

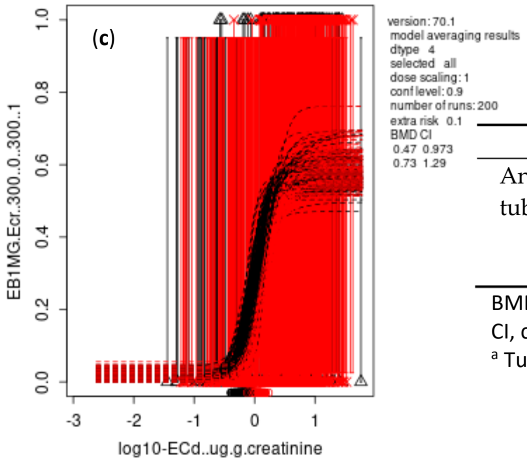

(d)

Quantal endpoint	BMDL	BMDU	U/L
An increase in prevalence of tubular dysfunction by 10%.			
Men	0.469	0.973	2.1
Women	0.733	1.29	1.8

BMDL and BMDU values of E_{Cd}/E_{cr} were as µg/g creatinine.
CI, confidence interval; U/L, BMDU/BMDL ratio.
[a] Tubular dysfunction was defined as $E_{\beta 2MG}/E_{cr} \geq 300$ µg/g creatinine.

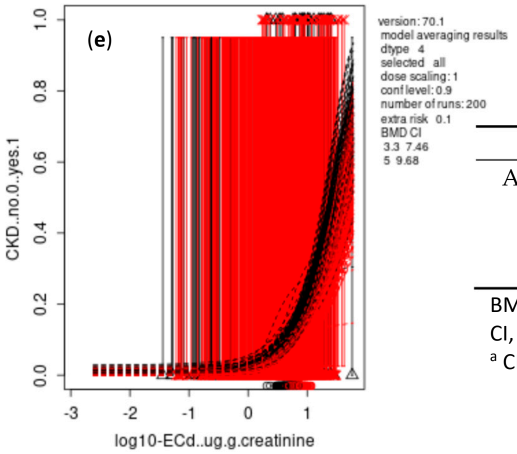

(f)

Quantal endpoint	BMDL	BMDU	U/L
An increase in prevalence of CKD [a] by 10%.			
Men	3.260	7.460	2.29
Women	4.980	9.680	1.94

BMDL and BMDU values of E_{Cd}/E_{cr} were as µg/g creatinine.
CI, confidence interval; U/L, BMDU/BMDL ratio.
[a] CKD was defined as eGFR ≤ 60 mL/min/1.73m^2.

Figure S4. Quantal dose-response analysis of E_{Cd}/E_{cr}. Bootstrap curves of model averaging of E_{Cd}/E_{cr} values associated 10% increases in the prevalence of tubular cell injury (**a**), tubular dysfunction (**c**), and CKD (**e**). BMDL and BMDU values of E_{Cd}/E_{cr} according to tubular injury (**b**), tubular dysfunction (**d**), and CKD (**f**).

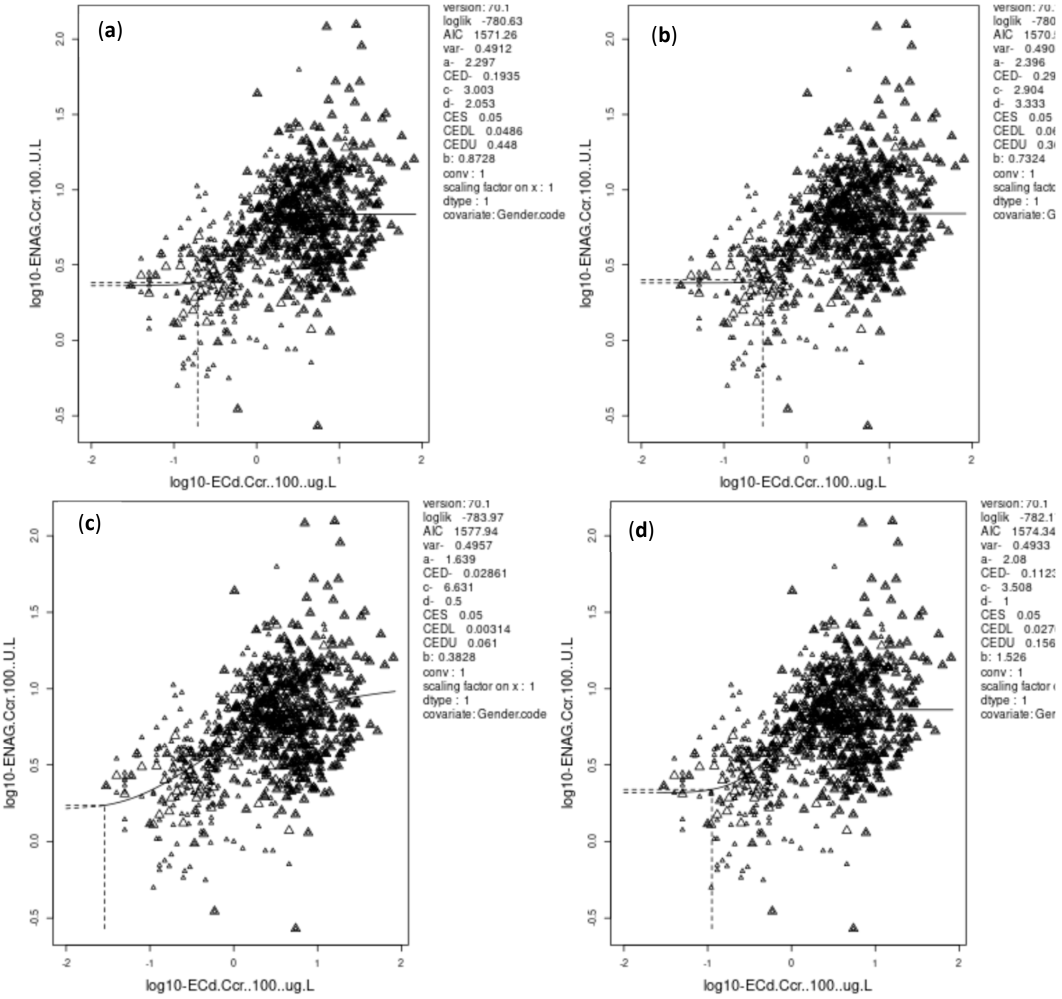

(e)

Continuous Endpoint	BMDL	BMDU	U/L
5% Increase of E_{NAG}/C_{cr}×100			
Men	0.067	0.394	5.88
Women	0.067	0.399	5.96

BMDL and BMDU values of E_{Cd}/C_{cr}×100 were as µg/L filtrate. CI, Confidence interval; U/L, BMDU/BMDL ratio.

Figure S5. Continuous dose-response analysis of E_{Cd}/C_{cr} and E_{NAG}/C_{cr}. Datasets were fitted to 4 dose-response models; an exponential model (**a**), Hill model (**b**), an inverse exponential model (**c**), and natural logarithmic model (**d**). Model averaging BMDL/BMDU values of E_{Cd}/C_{cr} associated with a 5% increase in E_{NAG}/C_{cr} (**e**).

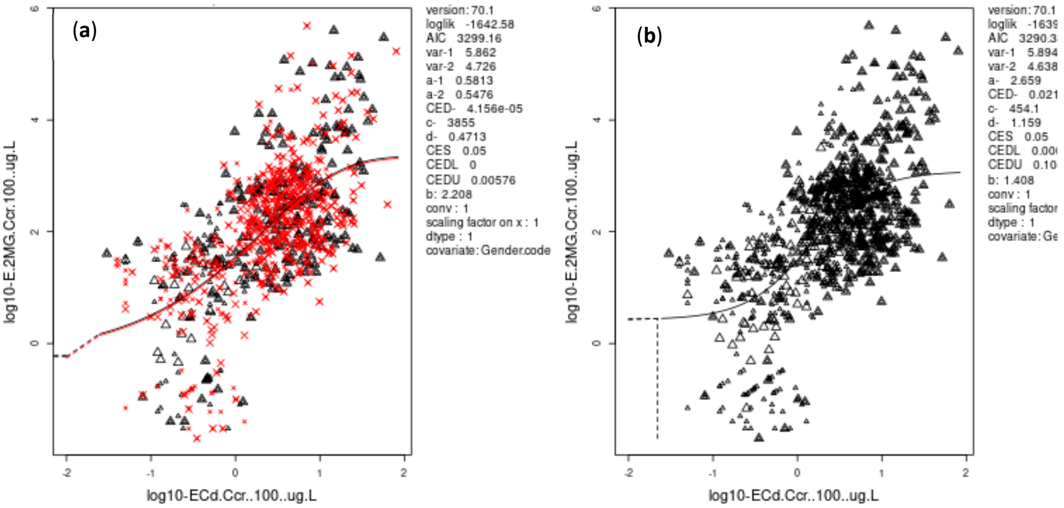

(c)

Continuous endpoints	BMDL	BMDU	U/L
5% Increase of $E_{\beta 2MG}/C_{cr}$ ×100			
Men	0.41×10^{-4}	0.038	929
Women	0.16×10^{-3}	0.040	255

BMDL and BMDU values of E_{Cd}/C_{cr}×100 were as µg/L filtrate. CI, Confidence interval; U/L, BMDU/BMDL ratio.

Figure S6. Continuous dose-response analysis of E_{Cd}/C_{cr} *vs*. $E_{\beta 2MG}/C_{cr}$. Datasets were fitted to an exponential model (**a**), and Hill model (**b**). Model averaging BMDL/BMDU values of E_{Cd}/C_{cr} associated with a 5% increase in $E_{\beta 2MG}/C_{cr}$ (**c**).

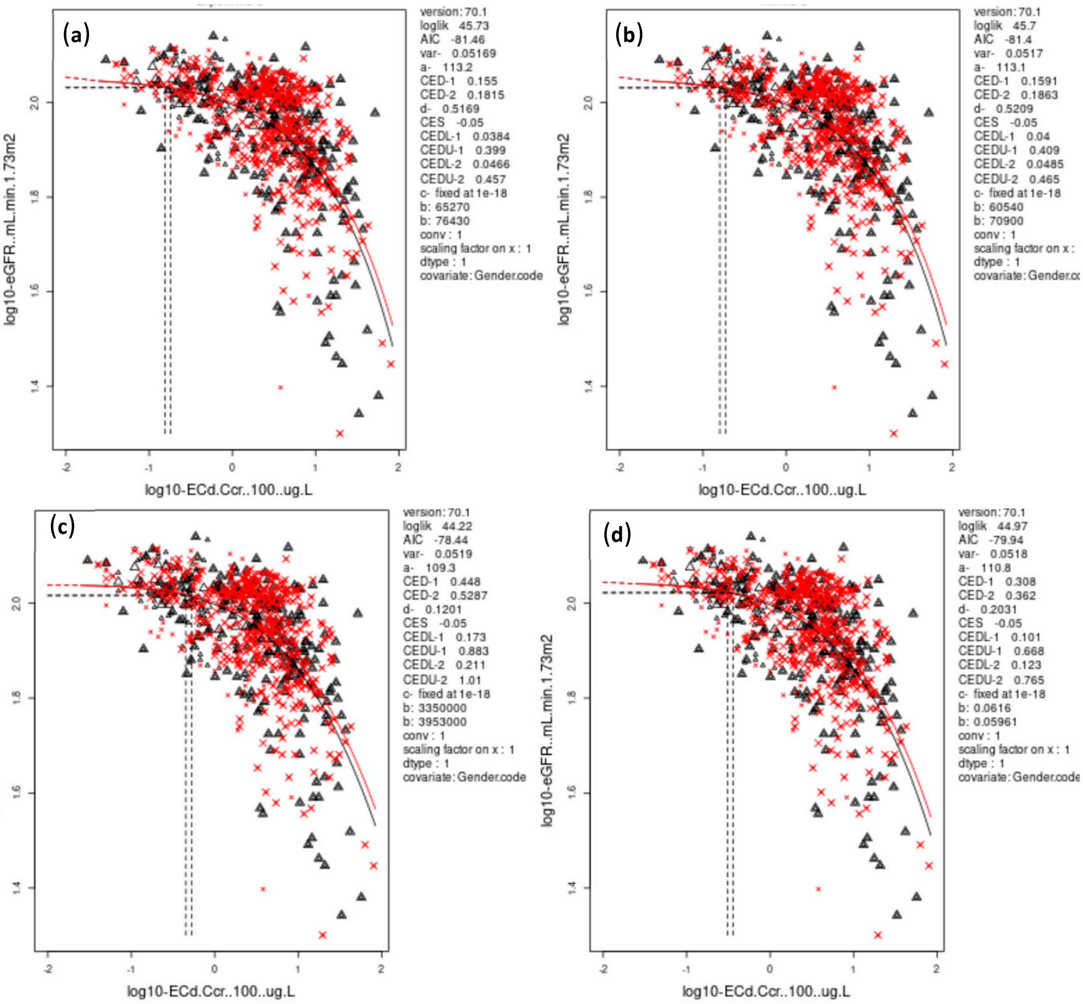

(e)

Continuous Endpoints	BMDL	BMDU	U/L
5% decrease of eGFR			
Men	0.080	0.714	8.92
Women	0.098	0.812	8.29

BMDL and BMDU values of E_{Cd}/C_{cr} ×100 were as μg/L filtrate. CI, Confidence interval; U/L, BMDU/BMDL ratio.

Figure S7. Continuous dose-response analysis of E_{Cd}/C_{cr} *vs.* eGFR. Datasets were fitted to 4 dose-response models; an exponential model (**a**), Hill model (**b**), an inverse exponential model (**c**), and natural logarithmic model (**d**). Model averaging BMDL/BMDU values of E_{Cd}/C_{cr} associated with a 5% decrease in eGFR (**e**).

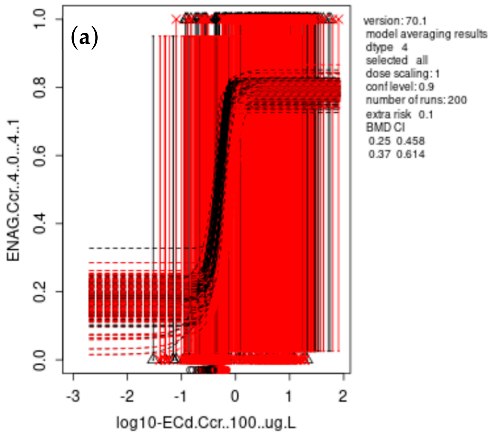

(b)

Quantal Endpoint	BMDL	BMDU	U/L
An increase in prevalence of tubular cell injury [a] by 10%.			
Men	0.254	0.458	1.80
Women	0.366	0.614	1.68

BMDL and BMDU values of E_{Cd}/C_{cr}×100 were as µg/L filtrate.
CI, confidence interval; U/L, BMDU/BMDL ratio.
[a] Tubular cell injury was defined as $E_{NAG}/C_{cr} \geq 4$ U/L filtrate.

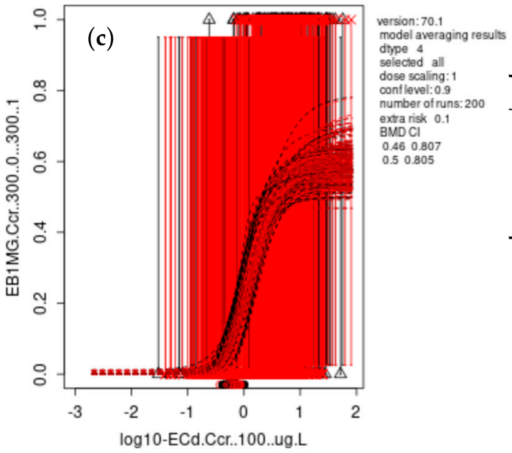

(d)

Quantal Endpoint	BMDL	BMDU	U/L
An increase in prevalence of tubular dysfunction [a] by 10%.			
Men	0.456	0.807	1.77
Women	0.500	0.805	1.61

BMDL and BMDU values of E_{Cd}/C_{cr} ×100 were as µg/L filtrate.
CI, confidence interval; U/L, BMDU/BMDL ratio.
[a]Tubular dysfunction was defined as $E_{\beta 2MG}/C_{cr}$×100 ≥300 µg/L filtrate.

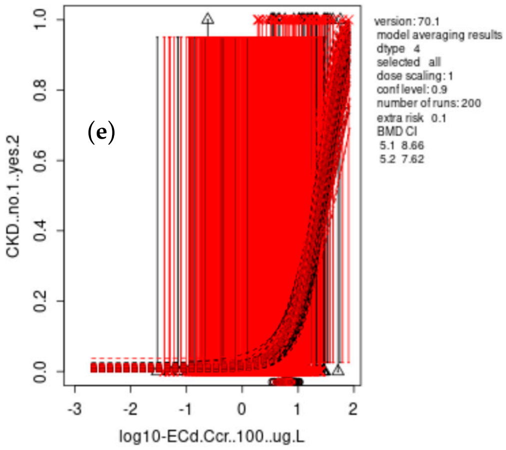

(f)

Quantal Endpoint	BMDL	BMDU	U/L
An increase in prevalence of CKD [a] by 10%.			
Men	5.060	8.660	1.71
Women	5.150	7.620	1.48

BMDL and BMDU values of E_{Cd}/C_{cr} ×100 were as µg/L filtrate.
CI, confidence interval; U/L, BMDU/BMDL ratio.
[a] CKD was defined as eGFR ≤ 60 mL/min/1.73m^2.

Figure S8. Quantal dose-response analysis of E_{Cd}/C_{cr}. Bootstrap curves of model averaging of E_{Cd}/C_{cr} values associated 10% increases in prevalence of tubular cell injury (**a**), tubular dysfunction (**c**), and CKD (**e**). BMDL and BMDU values of E_{Cd}/C_{cr} corresponded to 10% increases in prevalence of tubular injury (**b**), tubular dysfunction (**d**), and CKD (**f**).

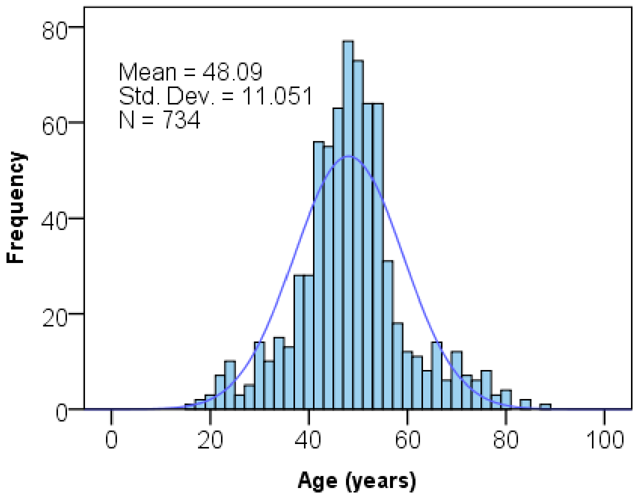

Figure S9. Histogram of age distribution of study subjects. There were 289 men and 445 women drawn from Bangkok (a low-exposure area) and from a Cd contaminated area of Mae Sot District (a high-exposure area) of Thailand [30]. The overall percentages of smokers, hypertension, and diabetes were 42.8%, 31.7% and 1.5%, respectively. The overall % of subjects with evidence for CKD, tubular cell injury and tubular dysfunction were 9%, 76.2%, and 39.8%, respectively.
